# A Mechanical Performance Study of Dual Cured Thermoset Resin Systems 3D-Printed with Continuous Carbon Fiber Reinforcement

**DOI:** 10.3390/polym15061384

**Published:** 2023-03-10

**Authors:** Md Atikur Rahman, Eric Hall, Luke Gibbon, Md Zahirul Islam, Chad A. Ulven, John J. La Scala

**Affiliations:** 1 Mechanical Engineering Department, College of Engineering, North Dakota State University, NDSU Dept 2490, P.O. Box 6050, Fargo, ND 58108, USA; 2 Combat Capabilities Development Command Army Research Laboratory, FCDD-RLW-MD, Aberdeen, MD 57401, USA

**Keywords:** additive manufacturing, 3D printing, continuous fiber-reinforced composites, carbon fiber, thermoset resin, photo curable resin, dual cured composites, mechanical properties, continuous fiber-reinforced thermoset 3D printing

## Abstract

Additive manufacturing (AM) is one of the fastest-growing manufacturing technologies in modern times. One of the major challenges in the application of 3D-printed polymeric objects is expanding the applications to structural components, as they are often limited by their mechanical and thermal properties. To enhance the mechanical properties of 3D-printed thermoset polymer objects, reinforcing the polymer with continuous carbon fiber (CF) tow is an expanding direction of research and development. A 3D printer was constructed capable of printing with a continuous CF-reinforced dual curable thermoset resin system. Mechanical performance of the 3D-printed composites varied with the utilization of different resin chemistries. Three different commercially available violet light curable resins were mixed with a thermal initiator to improve curing by overcoming the shadowing effect of violet light by the CF. The resulting specimens’ compositions were analyzed, and then the specimens were mechanically characterized for comparison in tensile and flexural performance. The 3D-printed composites’ compositions were correlated to the printing parameters and resin characteristics. Slight enhancements in tensile and flexural properties from some commercially available resins over others appeared to be the result of better wet-out and adhesion.

## 1. Introduction

Additive manufacturing (AM), also commonly referred to as 3D printing, has been around now for more than 30 years [1]. In the last decade, it has become one of the most exciting developments in manufacturing. AM has techniques available to produce parts utilizing both thermoplastic and thermoset materials [2,3]. Many techniques within manufacturing focus on the printing of thermoplastics, such as fused deposition modeling (FDM) [4,5,6]. However, because of their superior mechanical properties and thermal resistance, thermosets dominate the market in more industrial AM applications when using non-reinforced polymers. Processes to 3D print thermosets include stereolithography (SLA) [7,8,9], digital light processing (DLP) [10,11], and liquid deposition modeling (LDM) [1].

Composites of thermoset resins reinforced with continuous fiber are commonly used in high-performance composite applications, including aerospace, high-performance automobiles, boats, various sporting goods, medical, and wind turbines for renewable energy [12,13,14,15,16]. These are used in favor over thermoplastic composites for these high-performance applications because their lower initial viscosity allows for much higher fiber reinforcement content resulting in improved strength and stiffness and much higher resistance to creep. Yet, their initial low viscosity and challenges with cure on demand have resulted in limited development of commercial thermosetting composite printers and resulting parts. Although continuous fiber thermoplastic composite printing has certainly lagged behind unreinforced thermoplastic printing, such composite printers have succeeded, such as the Markforged Mark Two, and are used in industrial applications. The utilization of thermosetting composites has come to be of interest [17] because of its potential for higher-performance materials and because composites are highly tailorable to specifications. These industries and attributes represent a significant opportunity for the production of composites with AM. Recent advancements have produced the opportunity to achieve thermoset composite printing [18,19,20].

While there are multiple 3D printing techniques available to produce a thermoset composite, there are limitations. Fiber-reinforced thermoset composite 3D printing can be broken into two main categories, short fiber and continuous fiber [21,22,23,24,25]. Many printing techniques only allow for a short fiber composite to be produced [26]. In this form of printing, a thermoset resin is mixed with a batch of short fibers. This blend of material is then printed in its conventional way. One such way of achieving this print is through SLA printing. In this process, ultraviolet (UV) light is projected onto a flat surface of UV-curable resin. Projection of the UV light on the liquid resin initiates a polymerization reaction and solidifies the resin. This is often accomplished via a “bottom-up” process in which the print bed moves up out of a vat of resin. As the projection of an entire layer of the print is complete, the print bed moves, and the process repeats. This creates layers of the short fiber composite throughout the print. The fibers are not aligned in any way, yet they are influential in the final performance of the printed component. Although some mechanical properties are improved, significant jumps in performance are often not seen until a thermoset resin is combined with a continuous dry fiber [23,27,28].

Research has been carried out on short fiber and continuous fiber printing completed using fused filament fabrication (FFF) printing [29,30,31]. In this setup, a spool of filament, typically a thermoplastic, is set up and pushed through a heated extruder along with a continuous tow of CF [31,32]. For continuous fiber reinforcement in FFF printers, nozzle impregnation [33,34,35] and pre-impregnation [27,36,37] of polymers have been studied. Although successful, this form of printing was limited to thermoplastic filaments, such as ABS.

Continuous CF-reinforced thermoset composites were first 3D printed using a direct ink write (DIW)-based printing process by Hao et al. [24]. The major issue with DIW printing is the inability to print complex and tall structures [38]. Another method of AM that allows for the printing of continuous fiber-reinforced thermoset composites is liquid deposition modeling (LDM)-based printing which allows instant curing using violet light exposure [2,39]. In this form of printing, a photocurable resin is pumped from a vat through a nozzle. At this nozzle, there is an injection point where dry fiber is introduced to the resin. As the resin/fiber mix is deposited, a violet laser beam is focused on the bead of the material. This laser beam cures the resin enough to hold the composite bead in place. This process continues building layers on top of one another until the desired part has been processed. To complete curing, a thermal initiator can be used along with post-cure heating in an oven [24]. Pre-impregnating the dry fiber with the liquid thermoset resin and partially curing it before feeding it through the printing nozzle has also been studied [25]. In this study, printing parameters were optimized for maximizing the mechanical performance of 3D-printed thermoset composites.

Although this work has been in progress, minimal literature is in existence on continuous fiber-reinforced additive manufacturing. Furthermore, the applicability of commercially available SLA resin in this printing process has not been fully explored.

This study aimed to provide additional research into the processing and characterization of 1k tow carbon fiber reinforcement in multiple resins. Therefore, the purpose of this study was to demonstrate the violet laser beam-assisted 3D printing technology for continuous carbon fiber reinforced composite using commercially available thermoset resin systems. This was conducted with the goal of continuing the advancement of technologies relating to composite additive manufacturing.

In this study, an LDM technology-based 3D printer was designed and constructed to 3D print continuous CF-reinforced thermoset composite using laser beam assistance. Moreover, a custom nozzle was designed and manufactured to ensure in-nozzle impregnation of fiber with resin during the 3D printing process. These processes were used to perform a comparison of composite prints utilizing three separate commercially available thermoset resins, all combined with the same type of CF tow. To compare the performance of the resins, normalized tensile and flexural properties were investigated. In addition, the effectiveness of the dual cure system was confirmed by performing differential scanning calorimetry.

## 2. Materials and Methods

### 2.1. Materials

This study focused on utilizing a custom 3D printer that was adaptive to a wide range of materials. Three photo-curable acrylate resin systems were studied for print performance tests. Peopoly Nylon-Like Tough resin was obtained from Peopoly (Peopoly, Los Angeles, CA, USA) [40]. This was a mixture of a urethane diacrylate and an ethoxylated bisphenol-a diacrylate. Peopoly Deft resin was obtained from Peopoly (Peopoly, Los Angeles, CA, USA) [41]. This was a different formulation with urethane diacrylate and an ethoxylated bisphenol-a diacrylate. Liqcreate Strong-X resin was purchased from Liqcreate (Liqcreate, Utrecht, The Netherlands) [42]. The Liqcreate Strong-X formulation contained a mixture of dicyclopentadiene di methanol diacrylate, ethoxylated bisphenol-a diacrylate, and pentaerythritol tetra acrylate. These three resin systems came with a photoinitiator premixed from the manufacturer. A thermal initiator was added to each resin before using the resin for 3D printing. A Tenax 1K tow of CF from Teijin (Teijin Frontier, New York, NY, USA) was printed with all three resins [43]. The mechanical properties provided by the suppliers can be found in Table 1.

Utilizing a printer from a previous study [25], print parameters were selected and tested. Due to the opacity of CF, exposure to violet laser light did not achieve a full cure. A thermal initiator and post-curing process were added to enhance curing. Luperox P was mixed into each resin as a thermal initiator (tert-butyl peroxybenzoate 98%) from Sigma-Aldrich (St. Louis, MO, USA) [46]. The resin and thermal initiator were mixed for 2 min at 2500 rpm using a SpeedMixer (Hauschild Engineering, Model: SpeedMixer, Type: DAC-150 FVZ, Water camp, Hamm, Germany). Batches of 50 g resin were mixed at a time, and then the mixtures were degassed at −85 kPa using a vacuum chamber (VWR, Sheldon Manufacturing Inc., Model: 1415M, Cornelius, OR, USA).

### 2.2. 3D Printing of Continuous Fiber Reinforced Composite

**Figure 1:**
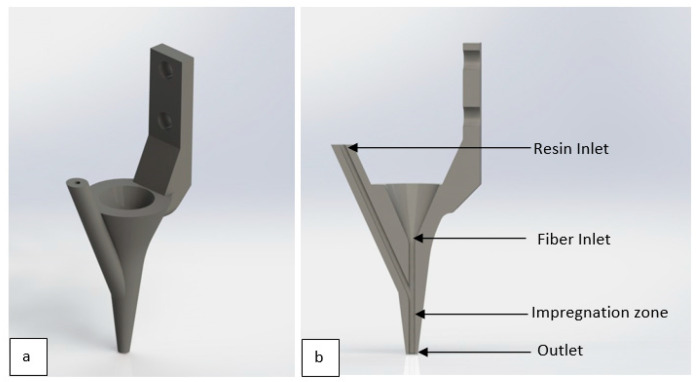
(**a**) Three-dimensional view of printing nozzle. (**b**) Cross-section of printing nozzle.

A 3D printer from a previous study [25] was utilized with a slightly modified form. In contrast to the original pre-impregnation process, a nozzle that incorporated in-nozzle resin impregnation into the fiber tow was used [25]. The printer was capable of printing continuous fiber-reinforced thermoset composites by utilizing nozzle resin impregnation. It was capable of varying multiple process parameters such as resin-to-fiber ratio and curing power. The system was developed with custom firmware to be adaptive for the control of modular process steps. The nozzle design is shown in Figure 1. The nozzles were 3D-printed with a Formlabs Form 2 printer (Formlabs, Sommerville, MA, USA) using Formlabs Tough 2000 resin [47]. The inner diameter of the impregnation zone of the nozzle was 1 mm in diameter and 15 mm in length. The resin was supplied to the nozzle system using a syringe pump.

**Figure 2:**
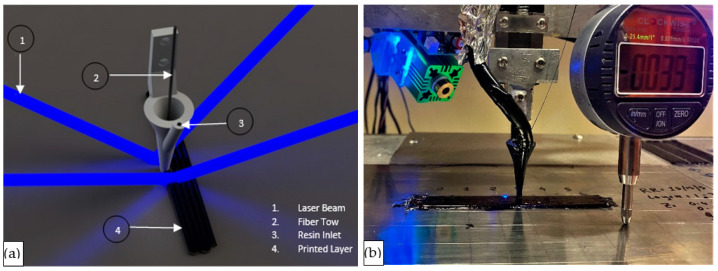
(**a**) Schematic of composite 3D printing process. (**b**) Actual printing process.

During the printing process, the fiber tow was pulled through the nozzle by the tension from the printed layers. The fiber tow was impregnated and encapsulated with the liquid resin in the impregnation zone, and the fiber tow carried this resin through the outlet tip as the layer was deposited. The deposited liquid resin in the printed layer was cured with violet lasers (Jolooyo, Wuhan, China, 405 nm wavelength, 150 mW output). A laser beam projected from each side perpendicular to the direction of nozzle movement followed the nozzle tip for each direction of the print-head’s back-and-forth movements. The schematic of the nozzle and laser beam arrangement is visualized in Figure 2. The laser projection point followed the nozzle tip with an offset distance of 5 mm. This offset was set to prevent the liquid resin from curing at the tip of the nozzle and obstruct material flow. Lasers were turned on and off depending on the print-head travel direction. The laser lighting sequence was set to always follow the nozzle in its track but never to illuminate the leading direction of the nozzle.

Composite rectangular bars were printed for mechanical property characterization. Each bar consisted of six layers of composite laminates reinforced with longitudinally unidirectional CF. The print parameters set for each resin sample group are listed in Table 2. The three resin systems that were utilized in this study exhibited different levels of light energy exposure requirement. As the laser output was set at a fixed value to accommodate for the varying requirement of sufficient light exposure, different resins were printed at different print speeds. With constant illumination power, a slower printing speed allowed a longer time for light exposure at any point of the dispensed resin, which increased the net amount of light energy at the exposed location. The print speed was adjusted by the trial-and-error method. If the printed lines did not stick properly to the printed layer, the print speed was slowed down to provide longer light exposure time. Longer exposure time produced higher levels of curing, and, as a result, uninterrupted printing was achieved. When the print speed was adjusted, the resin pumping rate was adjusted accordingly. Ideally, the target was to keep the ratio of print speed to resin pumping rate constant. Setting the speed and pumping rate as such should have produced the same fiber volume fraction ($V_{f}$) for the composites printed with different resins. However, because different resins had different viscous and adhesive properties, at a given print speed, each resin had a unique maximum allowable pumping rate without causing a backflow through the fiber entry port. The resin pumping rate was set below this point for the print speed set for each resin system. This caused the ratio of print speed to resin pumping rate to vary for different resin systems. This variation of speed to pumping rate ratio caused the variation of $V_{f}$ among the composites 3D-printed with different resin systems. The $V_{f}$ is discussed further in Section 3.2.

After completion of the printing, the printed bars were post-cured thermally in an oven (VWR, Sheldon Manufacturing INC, Model 1350FM-2, Cornelius, OR, USA) at 130 °C for 3 h. Though the printed specimens maintained good dimensional accuracy, the top surface of the printed specimens showed small ravines between the printed lines. At the corners, where the print lines made 180° turns, the CF tended to bend slightly upwards. To avoid interference between the printed layer and nozzle, these bumps at the ends were treated with mechanical abrasion. This was achieved during the printing process by utilizing a moving abrasive wheel on the printer. The printing process developed in this study can adapt to different types of fiber and resin materials and control additional process steps for the improvement of imperfections. The improvement of quality and properties of the printed specimens through optimization of process parameters are the focus of the continuation of this study.

### 2.3. Testing

#### 2.3.1. Differential Scanning Calorimetry (DSC)

The DSC test was conducted with 3D-printed composites and unreacted resin systems to ensure sufficient curing of the specimens after the post-curing stage. The amount of cure at the final product was investigated by comparing the heat flow curves of uncured and cured samples. These tests were conducted using a TA-modulated differential scanning calorimeter (DSC Q1000, TA Instruments, New Castle, DE, USA). The temperature range for the DSC test was 40 °C to 200 °C. Heat flow was measured at a temperature ramp set at 10 °C/min. Standard aluminum pans were used for testing the printed specimens, and hermetic aluminum pans were used for testing the unreacted resin mixtures.

#### 2.3.2. Density Testing

The densities of the composites were measured according to ASTM D792 [48]. Then, 20 (±0.05) mm × 18.5 (±0.3) mm × 2.8 (±0.2) mm coupons were weighed in air and immersed in distilled water using a precision digital scale (Veritas, Model: M254Ai, Veritas technologies Inc., Santa Clara, CA, USA). From the difference in these weights, the density of the composite was measured.

#### 2.3.3. Micro CT Testing

To measure the void content inside the composites, 20 (±0.05) mm × 18.5 (±0.3) mm × 2.8 (±0.2) mm printed coupons were CT-scanned. This test was carried out using GE microCT equipment (model: v|tome|x s, General Electronics, Boston, MA, USA).

#### 2.3.4. Burn-off Testing

Burn-off tests were conducted to determine the $V_{f}$ of the printed composites. Small sections of the printed composites were cut out and placed in the Lucifer Furnace (Model, RD4-KHE24, Warrington, PA, USA) to burn-off the matrix materials. The burn-off tests were carried out in reference to ASTM D3171 standards [49]. The composite was heated at 565 °C for 6 h in a nitrogen environment. Burn-off results were used to predict and normalize the mechanical properties of the printed specimens.

#### 2.3.5. Tensile Testing

The tensile properties of the 3D printed composites were tested by an Instron load frame (Model 5567, Norwood, MA, USA). Tensile test specimens were 150 mm long and had a cross-sectional area of 18.5 ± 0.3 mm wide and 2.8 ± 0.2 mm thick. The gauge length used was 100 mm. Tensile loads were measured using a 30 kN load cell, and strains were measured using a 25.4 mm extensometer. Tabs were attached to the printed composite using two-part epoxy. All tensile tests were conducted using the ASTM D3039 standard [50].

#### 2.3.6. Flexural Testing

Printed composites’ flexural properties were tested with a 3-point bending test using an Instron load frame (Model 5567, Norwood, MA, USA). Flexural tests were conducted to failure according to ASTM D7264 standards [51]. Flexural specimens had a cross-sectional area of 18.5 ± 0.3 mm wide and 2.8 ± 0.2 mm thick. The specimens were 100 mm in length. Flexural tests were performed using an Instron load frame (Instron, Series: 5567, Norwood, MA, USA) with a 30 kN load cell. According to the standard, the span-to-thickness ratio of the specimen under flexural load was set at 32:1.

## 3. Results and Discussion

### 3.1. DSC Testing

**Figure 3:**
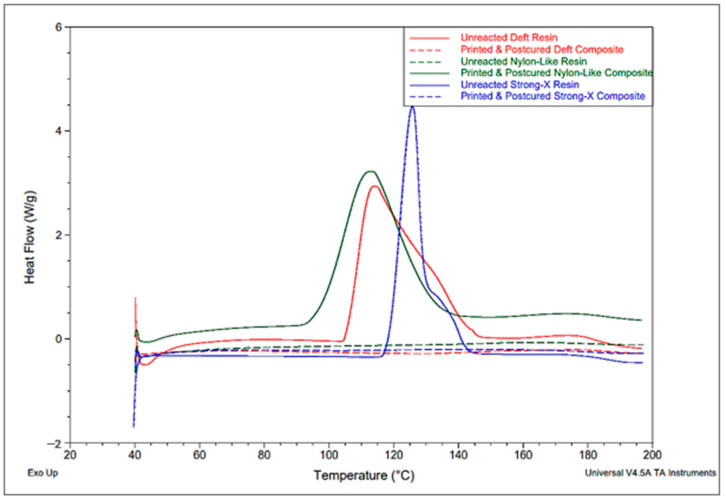
Heat flow curves of unreacted resins and post-cured composites during DSC tests.

Resin systems undergo an exothermic reaction during the curing process. The heat generated from this reaction was measured in the form of heat flow per unit mass during the DSC tests. When the resin was fully cured, it did not show any exothermic heat generation during the DSC tests. Figure 3 shows the plot of the exothermic heat flow of the resins and printed and post-cured composites. Peak heat flows were observed below 130 °C. Despite the residual heat of cure at ~175 °C for the DSC cured samples, the post-cured 3D-printed composite specimens did not show any exothermic heat flow. Thus, the post-cured specimens were considered sufficiently cured.

### 3.2. Compositional Testing and Void Analysis

Material constituent percentages were measured using a combination of Micro CT, fluid displacement density tests (immersion test), and burn-off tests. First, specimen sections were scanned with Micro CT to measure the void fraction inside the composites ($V_{v\;\left(int\right)}$).

After that, the densities of the composite specimens ($\rho_{c}$) were measured by weighing the specimens in air and immersed in distilled water at 23 °C. From these two weights, the densities of the composites were calculated. Using the mass difference before and after burning off the matrix materials, fiber volume fraction ($V_{f}$) was calculated with Equation (2). Here, *w_f_* and *w_m_* are the weight of fiber and matrix in the composite, respectively. $\rho_{f}$ and $\rho_{m}$ are the density of the fiber and the matrix, respectively. Here, using the resin density ($\rho_{r}$) as the matrix density would have been misleading as the resin volume shrank during the curing steps. Estimation of volumetric shrinkage of the resins from the manufacturer-provided linear shrinkage rate was not sufficient for a few reasons. Firstly, the resin was dual-cured with the help of an additional thermal initiator mixed into the formulations. Secondly, due to the nature of the line-wise 3D printing process, it was plausible that the resin could have shrunk at the fiber–matrix interface and at the interlaminar regions. Thirdly, the resin could also shrink within the matrix phase as well due to the dynamics of cross-linking. These factors could provide opportunities for internal voids to form. For these, matrix density was calculated using Equation (1). Here, $\rho_{c}$ is the composite’s density derived from the immersion tests. $m_{m}$ and $m_{f}$ are the mass fractions derived from the burn-off test. Two resin shrinkage factors $k_{d}$ and $k_{e}$ were defined using Equations (3) and (4), respectively. Here, $k_{d}$ is the volumetric shrinkage factor calculated from linear dimensional resin shrinkage percentage $s_{l}$. $k_{e}$ is calculated from experimental densities. The average composite densities were measured at 1.25, 1.17, and 1.14 gm/cm^3^ for the specimens with Deft, Nylon-Like, and Liqcreate resins. The *p*-value for composite density results was 7.92 × 10^−8^. The average matrix densities produced by Deft, Nylon-Like, and Strong-X resins were calculated to be 1.28, 1.27, and 1.25 gm/cm^3^, respectively. The difference in average matrix density was statistically significant, with a *p*-value of 1.03 × 10^−14^.

(1) $$\rho_{m}=\frac{m_{m}}{\frac{1-2V_{v\;\left(int\right)}}{\rho_{c\;}(1-V_{v\;\left(int\right)})}-\frac{m_{f}}{\rho_{f\;}}}$$

(2) $$V_{f}=\frac{\frac{w_{f}}{\rho_{f}}}{\frac{w_{f}}{\rho_{f\;}}+\frac{w_{m}}{\left(\rho_{m}\right)}+V_{v\;\left(int\right)}}$$

(3) $$k_{d}=1-{\left(\frac{s_{l}}{100}\right)}^{3}$$

(4) $$k_{e}=\frac{\rho_{m}}{\rho_{r}}\;$$

**Figure 4:**
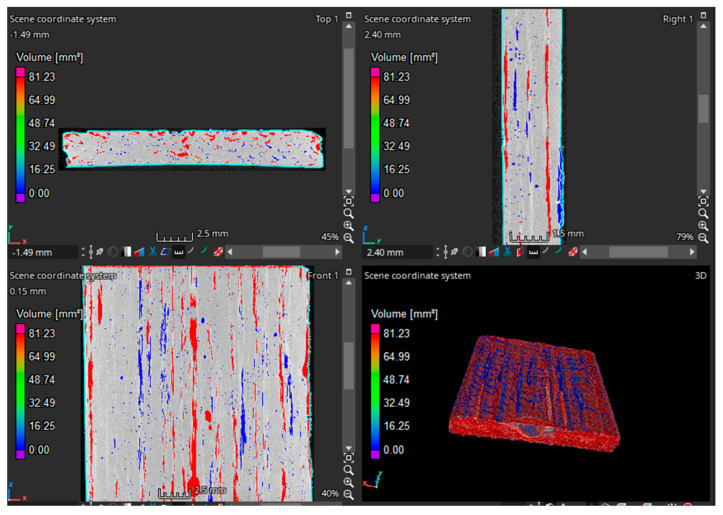
Micro CT imaging of composites.

**Figure 5:**
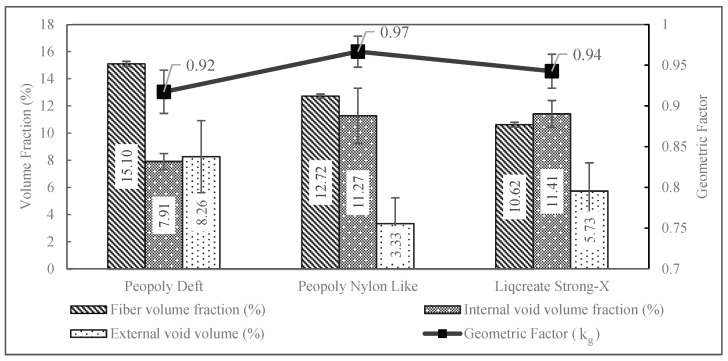
Composition of 3D-printed composite bars.

The internal void distributions and void volume factions of the composites were measured using Micro CT test results. The largest voids were aligned longitudinally in between the print lines. A sample void distribution from result Micro CT imaging can be seen in Figure 4. The average void percentages of the composites are visualized in Figure 5. Investigation of the void distribution showed the voids were more prevalent in the regions between adjacent print lines. No significant voids were observed to build up at the fiber–matrix interface.

The fiber mass fractions of the composites were calculated from the mass difference of the specimens before and after the burn-off test. As the burn-off was carried out in a nitrogen environment, it was possible that residual carbon from the matrix was present in the specimen after the burn-off. To validate the mass fraction from the burn-off test, mass flow percentages of the resin and fiber were calculated from the printing parameters using Equation (5). Here, $w_{f}^{\prime }$ = 0.074 gm/m is the weight of CF per unit length. From burn-off tests, average fiber mass fractions for Deft, Nylon-Like, and Liqcreate Resin composites were found to be 21.28%, 18.96%, and 16.21%, respectively. A *p*-value of 2.92 × 10^−12^ signified the statistical significance of the results. Mass fraction ($m_{f}$) predictions from Equation (5) were 19.13%, 17.07%, and 15.11%, respectively, for Deft, Nylon-Like, and Liqcreate resin composites. The close results of mass fraction calculations from the burn-off test and predictive mass fractions obtained from machine parameters validated the burn-off results. In this study, mass fractions obtained from burn-off results were utilized for further $V_{f}$ calculations. The composites’ fiber volume fractions and void fractions obtained from immersion tests, burn-off tests, and Micro CT tests are presented in Figure 5.

(5) $$m_{f}=\frac{\frac{w_{cf}^{\prime }S_{p}}{1000}}{\frac{{\dot{V}}_{r}\rho_{r}}{60}+\frac{w_{cf}^{\prime }S_{p}}{1000}}$$

The voids present in the composites profoundly influence the mechanical behavior of the printed composites. Analyzing the amount, distribution, and sources of the void content in the composites was important for the development and optimization of printable resins and their printing parameters. The significant difference between the densities of liquid resins and the densities of cured matrix indicated that shrinkage of the resins upon curing was a major factor behind void formation in the composites. The volume loss of resins upon curing could occur both inside the matrix or on the surfaces of printed lines. Slight trapped air at the printed line interface could grow due to resin shrinkage and thus grow large voids. With the simplifying assumption that voids are the volume loss due to resin shrinkage, the printed composites’ constituent percentages were predicted from print parameters using Equations (6)–(8). Different shrinking factors, *k*, were tested for composition simulation. The results are shown in Table 3.

(6) $$V_{f}=\frac{\frac{w_{f}^{\prime }S_{p}}{1000\;\rho_{f}}}{\frac{w_{cf}^{\prime }S_{p}}{1000\;\rho_{f}}+\frac{{\dot{V}}_{r}}{60}\;}$$

(7) $$V_{m}=\frac{\frac{{\dot{V}}_{r}}{60}k}{\frac{w_{f}^{\prime }S_{p}}{1000\;\rho_{f}}+\frac{{\dot{V}}_{r}}{60}\;}$$

(8) $$V_{v\;\left(int\right)}=\frac{\left(1-k\right)\frac{{\dot{V}}_{r}}{60}}{\frac{w_{f}^{\prime }S_{p}}{1000\;\rho_{f}}+\frac{{\dot{V}}_{r}}{60}\;}$$

$V_{f}$ predictions for each predictive model were very close to experimental values. On the contrary, the matrix volume fraction $V_{m}$ was higher than the experimental values when predicted with no shrink condition. When shrinkage factors were coupled in the predictive model, compositional prediction closely resembled the actual results. These supported the contribution of resin shrinkage towards void formation. From the data presented in Table 1 and Table 3, it was noticed that the resin with the lowest viscosity produced composites with void content lower than the predicted void content. This could have happened due to the ease of mobility of materials and void during the matrix formation. This mobility could have allowed the matrix to be formed in a compact fashion and ejected some of the voids through the open surfaces. Further study is required to confirm this effect. If more of the volume shrinkage for this resin happened through the surface, then the composites with this resin should have the lowest geometric factor (discussed in the following paragraph). This is supported by the highest amount of external void (surface voids or ravines) volume.

**Figure 6:**
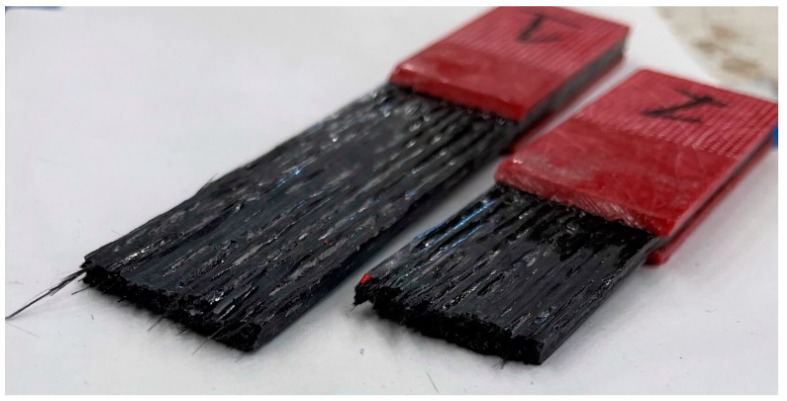
Tensile specimen failure section.

The printed composite specimen did not have a smooth surface finish. The presence of longitudinal ravines can be observed at the top surface of the specimen in Figure 6. As the specimen dimensions were measured using slide calipers, the cross-sectional area calculations had slight errors. A geometric factor $k_{g}$ was introduced to filter out the missing external volumes from material characterization. To do so, specimens of length l = 10 ± 0.05 mm were cut with precisely measured length. Then, the volume $V_{1}$ was calculated from calipers measured widths and thicknesses. With immersion testing being similar to density testing, another measurement of volume $V_{2}$ was performed for the same specimens. The difference between these two volumes provided the measure of external void content present in the general measurements. The percentage of external void content is shown in Figure 5. The expression for $k_{g}$ is provided by Equation (9). By averaging volume ratios of known lengths, Equation (9) provided the ratio of cross-sectional area and thus provided the factor for cross-sectional area correction.

(9) $$k_{g}=\frac{V_{2}}{V_{1}}$$

As the general practice for obtaining cross-sectional dimensions of specimens is to use calipers, the expected strength and modulus value from these measurements would be slightly higher than an actual estimation. Thus, factoring $k_{g}$ in the predictive modeling could rectify the result towards more realistic approximations. Furthermore, it is worth mentioning that $k_{g}$ is dependent on the geometry of the test specimens. So, specimens with different widths and thicknesses would yield a different value of $k_{g}$. Specimens with a larger cross-sectional area should yield a smaller value of $k_{g}.$

### 3.3. Tensile Testing

Tensile specimens produced clean brittle failure modes, as expected from a carbon fiber composite utilizing a brittle matrix. The stress–strain data from the tensile test were plotted, and tensile elastic modulus, E, was calculated. Figure 6 illustrates the failures typical with all the specimens in this study. Figure 6 also exhibited the quality of the external surface of the printed specimen. As seen in Figure 6, the external surface of printed specimens contains voids in-between adjacent fiber beads. The volume loss due to the surface roughness was captured by the geometric factor $k_{g}$. However, that roughness due to void content can be minimized by optimizing the resin rate further, which could be a potential field to explore further in future.

**Figure 7:**
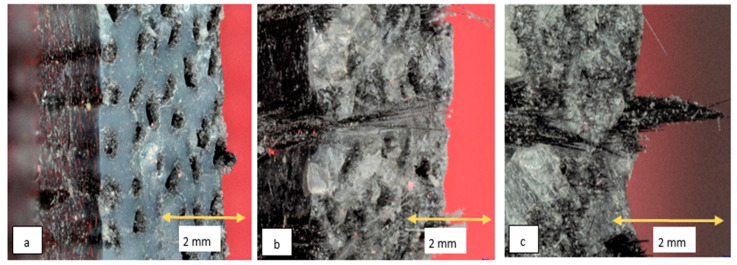
Microscopic image of tensile fracture surfaces. (**a**) Liqcreate Strong-X Composite, (**b**) Peopoly Nylon-Like Composite, and (**c**) Peopoly Deft Composite.

The tensile fracture surfaces of the composites of each resin type were investigated with microscopic imaging. Microscopic imaging was conducted using a Keyence VHX microscope (Keyence, Model: VHX, Osaka, Japan). The fracture surfaces of the tensile specimens are shown in Figure 7. Distinct differences between the fracture surfaces were observed among the composites. Though all three composites exhibited some degree of fiber pullout, the composite with Liqcreate-X showed maximum fiber pullout tendency. Moreover, the Liqcreate-X composite matrix failed perpendicular to the loading direction with an almost flat fracture surface (indicating brittle matrix failure). On the other hand, the two composites with Peopoly resin showed rough failure surfaces in the matrix (indicating some ductility in the matrix). The Peopoly Nylon-Like and the Peopoly Deft composites also exhibited higher fiber adhesion and better fiber impregnation compared to the Liqcreate composites. Table 1 shows that the Liqcreate Strong-X matrix has the least tensile elongation at failure. This complies with the microscopic fracture surface images.

**Figure 8:**
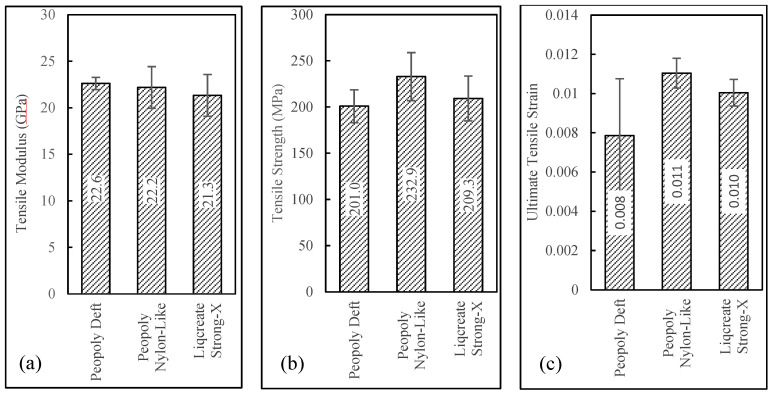
(**a**)Tensile modulus, (**b**) tensile strength, and (**c**) ultimate tensile strain.

Tensile results can be seen in Figure 8. From this plot, it was observed that tensile strength of 232.9 MPa was displayed by the composites printed with Peopoly Nylon-Like resin. Though the tensile strength and strain were slightly different for the three resins, the tensile modulus was similar, around 21 GPa for all three resin systems. The ultimate tensile strain of composites printed with the Peopoly Deft resin system was lower compared to the other resin systems. However, the wide error bars for this resin system indicated the possibility of statistical deviation. Single-factor ANOVA tests among the resin groups revealed *p*-values of 0.11, 0.31, and 0.004 for tensile strength, modulus, and strain values, respectively. This indicated that the slight difference in mean values of tensile strength and modulus was not statistically significant. Nevertheless, the composite specimens, printed with different resins, had a statistically significant difference in fiber volume fraction values. For this reason, the tensile strength and modulus values were normalized against $V_{f}$ and $V_{v}$ values to further investigate the difference in the tensile performance of the specimens printed with different resins. Detailed discussion about the normalization of tensile properties is provided in the following sections.

**Figure 9:**
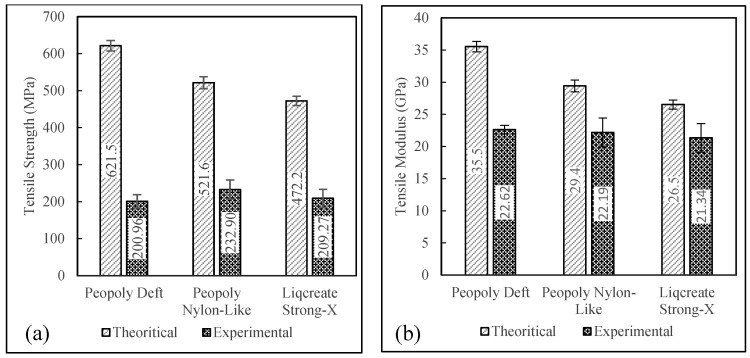
(**a**) Theoretical vs. experimental tensile modulus and (**b**) theoretical vs. experimental tensile strength.

The mechanical properties of fiber and the matrix materials control the tensile properties of the 3D printed composites. The properties of the composites could be altered by varying the $V_{f}$. Using the $V_{f}$ values from Figure 5, theoretical tensile strength and modulus were modeled by the rule of mixture (ROM) with slight modification. The cross-sectional area corrected ROM model is expressed in Equations (10) and (11). Here, $\sigma_{cu}$, $\sigma_{fu}$, and $\sigma_{mu}$ are the tensile strengths of the composite, fiber, and matrix, respectively. $E_{c},\;E_{f}$, and $E_{m}$ are the tensile moduli of the composite, fiber, and matrix, respectively. In this model, the geometric factor $k_{g}$ was included to adjust the actual cross-sectional area that resembles the printed specimens. Figure 9 shows a comparison of experimental vs. theoretically predicted tensile properties.

(10) $$\sigma_{cu}=k_{g}\left(\sigma_{fu}V_{f}+\sigma_{mu}V_{m}\right)$$

(11) $$E_{c}=k_{g}(E_{f}V_{f}+E_{m}V_{m})$$

Figure 9 shows that, though the ROM predicted different tensile moduli for different material combinations, the experimental tensile moduli were almost similar. As noted in Section 3.2, the three different material combinations yielded composites with different $V_{f}$. The ratios of experimental to theoretical tensile moduli of Deft, Nylon-Like, and Strong-X composites are 0.64, 0.74, and 0.80, respectively. The ratios of experimental to theoretical tensile strengths are 0.32, 0.45, and 0.44 for the composites with Deft, Nylon-Like, and Strong-X resins, respectively.

Here, the ROM model was used for predicting the theoretical tensile properties of unidirectional fiber composites. Although this is a simple, straightforward model, this model almost always overestimates the tensile properties of composites. Although it is not uncommon to see 20% differences in performance vs. modeling, the severely underperforming experimental data was unexpected. In an effort to analyze this further, the strain-to-failure values of each component from Table 1 were taken into consideration. When looking at the ratio of experimental strain-to-failure compared to expected strain-to-failure (from 1.7% fiber ultimate strain) and adjusting the ROM accordingly, it was found that the expected maximum stress values at roughly 1% strain to failure were within 35% of the experimental findings. While this does not explain the cause of failure, it does provide an indication that failure at this point was within estimated performance values at 1% strain. Further investigation is necessary. However, factors including, but not limited to, the effect of the thermal initiator, the potential for post-processing stresses, and fiber alignment could be considered. Certainly, the presence of surface defects that cause ravines in the surface of the sample reduces the actual thickness of the part. The thickness was corrected with $k_{g}$, which did not rule out the effect of local stress concentrations, which could have a detrimental effect on the tensile performance.

**Figure 10:**
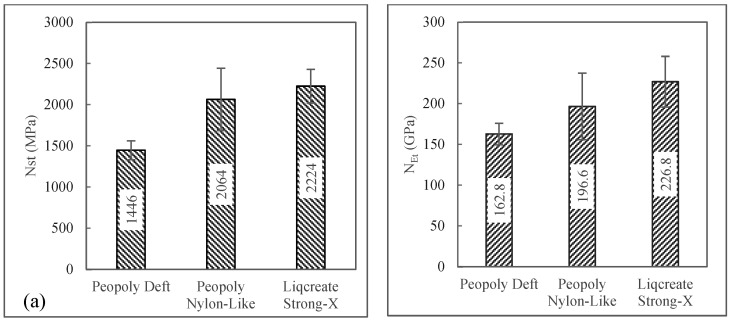
(**a**) Normalized tensile strength. (**b**) Normalized tensile modulus.

ROM is not the only model that predicts tensile performance. The model does not consider many factors such as distribution of voids, debonding, delamination, interfacial shear, the statistical distribution of fiber fracture, etc. [52]. To better understand the tensile behavior of the unidirectional fiber composites, different models could be studied, such as Cox’s shear lag model [53], Curtin’s global load sharing model [54], Rosen’s weakest link theory [55], Zhou and Wagner’s load redistribution model [56], Liao’s model [57], and many more. When composite properties are fiber dominated, almost all the models predict an increase in tensile strength and modulus for increased $V_{f}$ of fiber-dominated unidirectional composites. The Opposite is true for void concentration level. For resin matrix performance comparison, we can introduce a simplified normalized tensile property parameter respective to $V_{f}\;\mathrm{and}\;V_{v}.$ The normalized tensile strength and modulus ($N_{St}$ and $N_{Et})$ are described by Equations (12) and (13), respectively. Here, $\sigma_{cu}$ and $E_{c}$ are the experimental tensile strength and modulus of composites, respectively. These normalized properties can capture the performance of the matrix for a fixed fiber in the composite. $N_{St}$ for the Deft, Nylon-Like, and Strong-X based resin systems are shown in Figure 10. From this representation, the comparative tensile performance could be evaluated more clearly. It showed that for a fixed $V_{f}$ and $V_{v}$ among the three material combinations, the composites with the Liqcreate Strong-X resin system would show the greatest tensile properties. The results do not scale inversely with viscosity, indicating that void concentration and defects are not the reason for this effect. On the other hand, the results do scale with the strength and modulus of the neat resin, indicating that there is a coupling inefficiency between compliant resins and rigid fibers relative to rigid resins and rigid fibers. Indication towards the coupling inefficiencies can be observed from the microscopic images shown in Figure 7.

(12) $$N_{St}=\frac{\sigma_{cu}}{V_{f}\left(1-V_{v}\right)\;}$$

(13) $$N_{Et}=\frac{E_{c}}{V_{f}\left(1-V_{v}\right)\;}$$

### 3.4. Flexural Testing

**Figure 11:**
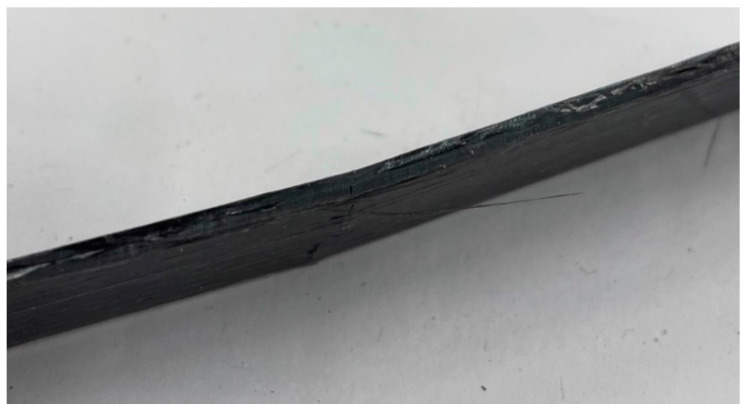
Failed flexural specimen.

Flexural specimens tested in this study using the 32:1 span-to-thickness ratio failed nearly identically. The failure mode was a bottom-face tensile failure with no signs of crushing the top face. A typical failed flexural specimen can be seen in Figure 11. Flexural stress, $\sigma_{f}$, was calculated from Equation (14). In this equation, $\sigma_{f}$ is stress in the outer fibers at the midpoint, *P* is the load given at a point on the load–deflection curve, *L* is the supported span, *b* is the width of the beam, and *d* is the depth of the beam.

**Figure 12:**
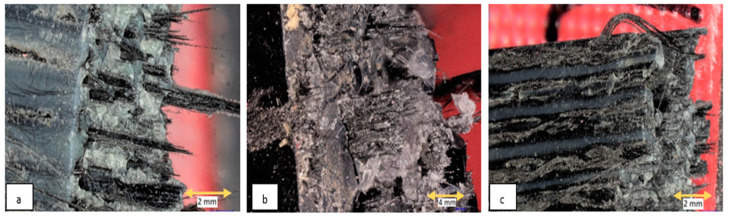
Bottom tensile fracture surface of flexural test specimens. (**a**) Liqcreate Strong-X Composite, (**b**) Peopoly Nylon-Like Composite, and (**c**) Peopoly Deft Composite.

Microscopic investigation of the tensile region of the flexural specimens revealed different amounts of fiber pullout among the composites. The flexural failure surfaces are presented in Figure 12. In these images, it can be observed that the Liqcreate composite exhibited the highest amount of fiber pullout. The length of pulled-out fiber was also the longest for this composite. The presence of some dry fibers was also observed in the Liqcreate composites.

Equation (15) was used for calculating flexural strain, $\varepsilon_{fl}$, at the outer surface of the sample. Here, *D* is the max deflection at the center of the beam, and *d* is the depth of the beam.

Along with the flexural stress and strain, flexural chord modulus $E_{fl}$ was calculated using Equation (16). In this equation, $\sigma_{fl1}$ and $\sigma_{fl2}$ are flexural stresses measured at predefined points on the load–deflection curve, and $\varepsilon_{fl1}$ and $\varepsilon_{fl2}$ are flexural strains measured at predefined points on the load–deflection curve.

(14) $$\sigma_{fl}=\frac{3PL}{2bd^{2}}$$

(15) $$\varepsilon_{fl}=\frac{6Dd}{L^{2}}$$

(16) $$E_{fl}=\frac{\sigma_{fl1}-\sigma_{fl2}}{\varepsilon_{fl1}-\varepsilon_{fl2}}$$

**Figure 13:**
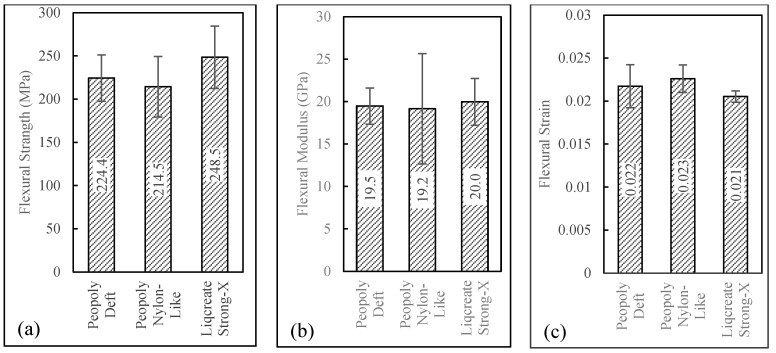
(**a**) Flexural strength, (**b**) flexural modulus, and (**c**) flexural strain.

Flexural results can be seen in Figure 13. Here, it was observed that composites with three different matrices show very similar flexural properties. Single-factor ANOVA *p*-values among the different composites were 0.28 and 0.95 for flexural strength and flexural modulus, respectively. As the P-values were greater than 0.05, the variation of mean flexural properties among different resin composites was not statistically significant. The Strong-X-based matrix composites showed the highest average flexural strength of 249 MPa, though this composite had the lowest average $V_{f}$ and highest $V_{v}$ among the composites. To better display the effect of matrix materials on the flexural properties of the 3D-printed composites, once again, flexural strength and modulus were normalized with $V_{f}$ and $V_{v}$. This was carried out for flexural strength and modulus according to Equations (17) and (18), respectively. Here, $S_{fl}$ and $E_{fl}$ are the experimental flexural strength and modulus of the composites.

(17) $$N_{Sfl}=\frac{S_{fl}}{V_{f}\left(1-V_{v}\right)\;}$$

(18) $$N_{Efl}=\frac{E_{fl}}{V_{f}\left(1-V_{v}\right)}$$

**Figure 14:**
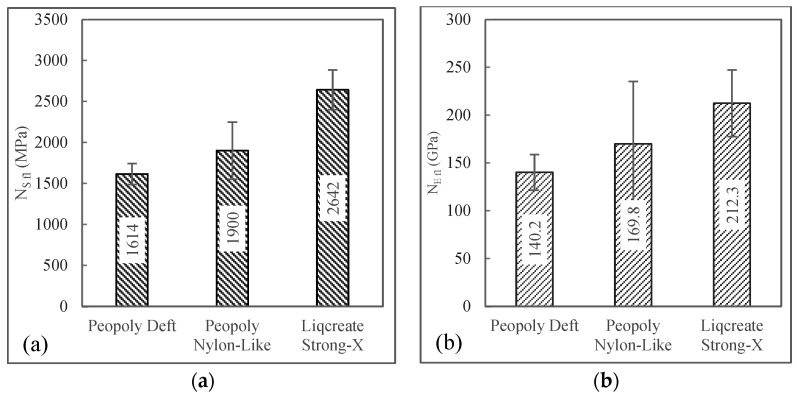
(**a**) Normalized flexural strength and (**b**) normalized flexural modulus.

The normalized flexural properties are visualized in Figure 14. This data set showed that with the same fiber volume fraction, flexural strength and modulus were the highest for the Strong-X-based resin system. It was also observed that the composites with the Nylon-Like resin system underperformed in the flexural test compared to the tensile tests. This could be due to inefficient fiber matrix coupling.

## 4. Conclusions and Future Recommendations

In this study, the applicability of commercially available thermoset resin systems into continuous fiber reinforcement composite 3D printing and the mechanical performance of the printed composites were explored. This study successfully developed the violet light-assisted LDM-based 3D printing technology for continuous CF-reinforced composites using three commercially available thermoset resin systems (Liqcreate- X, Peopoly Nylon-Like, and Peopoly Deft). To ensure full curing of resin, post-curing of the printed specimen was carried out thermally in a high-temperature oven.

The constituent compositions of printed composites were fully characterized. According to the burn-off test results of the printed specimens, each resin system achieved varying fiber volume fractions. When each system was normalized for this, the resin system for 3D-printed composites with the greatest mechanical properties was identified as Liqcreate Strong-X. In the findings, tensile properties were found to be less than anticipated. It is the authors’ belief that factors within post-curing, the printing process, and the fiber–resin interaction contribute to the large discrepancy between experimental and ROM-predicted values. Surface defects and better coupling efficiency between rigid resin and fiber vs. compliant resin and fiber are also subjects of further studies.

The source of void formation was investigated. Even though the void could have formed from a few sources, a positive correlation was observed between the shrinkage rate of the resins and the void percentage that it incorporated into the composites. This could be a very important factor to consider for the optimization of the print parameters and requires further in-depth studies for conclusive understanding.

It was shown that although the resins were developed and optimized for SLA 3D printing, they can be altered and used for 3D printing continuous carbon fiber reinforced composite. Investment of resources in purposefully developed resin systems for this composite 3D printing technology could yield even higher-performing printed composites. Furthermore, DSC results showed that the designed dual cure system was sufficiently effective to ensure full cure of the resin matrix. As carbon fiber–epoxy resins are being widely adopted in numerous industries as high-performance composites, developing a dual curable (light and thermal) high-performance resin system and carbon fiber sizing optimized for that specific resin can produce 3D-printed composites which can exhibit mechanical properties significantly higher than any currently available commercial 3D printing system.

In future work, it would be of interest to identify and determine to what extent variables such as print speed, nozzle angle, cure temperature, thermal expansion, void content, and fiber alignment all affect mechanical performance.

## Figures and Tables

**Table 1:** Mechanical properties of composite components [40,41,42,43,44,45].

Properties	Peopoly Nylon-Like Tough	Peopoly Deft	Liqcreate Strong-X	Tenax
Tensile strength (MPa)	62	35	60–84	4100
Tensile modulus (GPa)	2.05	0.75	3.1–3.4	240
Elongation (%)	44	6	3–6	1.7
Density (gm/cm^3^)	1.12	1.12	1.12	1.77
Viscosity (cps at 25 °C)	780	105	550	-
Linear shrinkage after curing (%) $s_{l}$	6.5	6.5	1.5	-

**Table 2:** Print parameters.

Layer Thickness	0.45 ± 0.03 mm
Line Spacing	1.0 mm
Nozzle Diameter	1.0 mm
Post-cure (130 °C)	3 h
Print Speed, $S_{p}$ (mm/min)	Peopoly Deft: 550 Peopoly Nylon-Like: 270 Liqcreate Strong-X: 310
Resin Pumping Rate, ${\dot{V}}_{r}$ (cc/hr)	Peopoly Deft: 9.5 Peopoly Nylon-Like: 5.2 Liqcreate Strong-X: 6.9

**Table 3:** Predicted composite compositions vs. actual compositions.

Matrix	Void Source	$V_{f}\;(\%)$	$V_{m}\;(\%)$	$V_{v\;\left(int\right)}\;(\%)$
**Peopoly Deft**	k = 1 (no shrinkage)	13.02	86.98	0
	$k=k_{d}\;$, void = shrunk volume	13.02	71.10	15.88
	$k=k_{e}$, void = shrunk volume	13.02	75.86	11.12
	Actual composition *	15.10	76.99	7.91
**Peopoly Nylon-Like**	k = 1 (no shrinkage)	11.52	88.47	0
	$k=k_{d}$, void = shrunk volume	11.52	72.32	16.16
	$k=k_{e}$, void = shrunk volume	11.52	78.11	10.37
	Actual composition *	12.72	76.00	11.27
**Liqcreate Strong-X**	k = 1 (no shrinkage)	10.13	89.87	0
	$k=k_{d}$, void = shrunk volume	10.13	85.88	3.99
	$k=k_{e}$, void = shrunk volume	10.13	80.65	9.22
	Actual composition *	10.62	77.97	11.41

* Actual composition of the composite was calculated from the micro-CT, Burn-off, and density test results using Equations (1) and (2).

## Data Availability

The data presented in this study are available on request from the corresponding author.

## References

[1] Horn T.J., Harrysson O.L.A. (2012). Overview of Current Additive Manufacturing Technologies and Selected Applications. Sci. Prog..

[2] Tamez M.B.A., Taha I. (2020). A review of additive manufacturing technologies and markets for thermosetting resins and their potential for carbon fiber integration. Addit. Manuf..

[3] Lee J., Lee H., Cheon K.-H., Park C., Jang T.-S., Kim H.-E., Jung H.-D. (2019). Fabrication of poly(lactic acid)/Ti composite scaffolds with enhanced mechanical properties and biocompatibility via fused filament fabrication (FFF)–based 3D printing. Addit. Manuf..

[4] He F., Ning H., Khan M. (2023). Effect of 3D Printing Process Parameters on Damping Characteristic of Cantilever Beams Fabricated Using Material Extrusion. Polymers.

[5] Liu J., Naeem M.A., Al Kouzbary M., Al Kouzbary H., Shasmin H.N., Arifin N., Razak N.A.A., Abu Osman N.A. (2023). Effect of Infill Parameters on the Compressive Strength of 3D-Printed Nylon-Based Material. Polymers.

[6] Bandinelli F., Peroni L., Morena A. (2023). Elasto-Plastic Mechanical Modeling of Fused Deposition 3D Printing Materials. Polymers.

[7] Valencia L.M., Herrera M., de la Mata M., Hernández-Saz J., Romero-Ocaña I., Delgado F.J., Benito J., Molina S.I. (2022). Stereolithography of Semiconductor Silver and Acrylic-Based Nanocomposites. Polymers.

[8] Choi Y., Yoon J., Kim J., Lee C., Oh J., Cho N. (2022). Development of Bisphenol-A-Glycidyl-Methacrylate- and Trimethylolpropane-Triacrylate-Based Stereolithography 3D Printing Materials. Polymers.

[9] Ávila-López M.A., Bonilla-Cruz J., Méndez-Nonell J., Lara-Ceniceros T.E. (2022). Strong and Lightweight Stereolithographically 3D-Printed Polymer Nanocomposites with Low Friction and High Toughness. Polymers.

[10] Chaudhary R., Akbari R., Antonini C. (2023). Rational Design and Characterization of Materials for Optimized Additive Manufacturing by Digital Light Processing. Polymers.

[11] Arias-Ferreiro G., Lasagabáster-Latorre A., Ares-Pernas A., Ligero P., García-Garabal S.M., Dopico-García M.S., Abad M.-J. (2022). Lignin as a High-Value Bioaditive in 3D-DLP Printable Acrylic Resins and Polyaniline Conductive Composite. Polymers.

[12] Košir T., Slavič J. (2023). Modeling of Single-Process 3D-Printed Piezoelectric Sensors with Resistive Electrodes: The Low-Pass Filtering Effect. Polymers.

[13] Ciobanu R.C., Schreiner C., Aradoaei M., Hitruc G.E., Rusu B.-G., Aflori M. (2023). Characteristics of Composite Materials of the Type: TPU/PP/BaTiO_3_ Powder for 3D Printing Applications. Polymers.

[14] Egan P.F., Khatri N.R., Parab M.A., Arefin A.M.E. (2022). Mechanics of 3D-Printed Polymer Lattices with Varied Design and Processing Strategies. Polymers.

[15] Lee H., Kim Y., Kim J., Moon S.Y., Lee J.U. (2022). Consecutive Ink Writing of Conducting Polymer and Graphene Composite Electrodes for Foldable Electronics-Related Applications. Polymers.

[16] Zhu Y., Tang T., Zhao S., Joralmon D., Poit Z., Ahire B., Keshav S., Raje A.R., Blair J., Zhang Z. (2022). Recent Advancements and Applications in 3D Printing of Functional Optics. Addit. Manuf..

[17] Simpson P., Holthaus M.J., Gibbon L., Ulven C.A. (2021). Short Glass Fiber Reinforced Perspective Chapter: Composites Manufactured by Stereolithography. IntechOpen.

[18] Singh S., Ramakrishna S., Berto F. (2020). 3D Printing of polymer composites: A short review. Mater. Des. Process. Commun..

[19] Bekas D.G., Hou Y., Liu Y., Panesar A. (2019). 3D printing to enable multifunctionality in polymer-based composites: A review. Compos. Part B Eng..

[20] Mahmood A., Akram T., Chen H., Chen S. (2022). On the Evolution of Additive Manufacturing (3D/4D Printing) Technologies: Materials, Applications, and Challenges. Polymers.

[21] Wang B., Zhang Z., Pei Z., Qiu J., Wang S. (2020). Current progress on the 3D printing of thermosets. Adv. Compos. Hybrid Mater..

[22] He X., Ding Y., Lei Z., Welch S., Zhang W., Dunn M., Yu K. (2021). 3D printing of continuous fiber-reinforced thermoset composites. Addit. Manuf..

[23] Hao W., Liu Y., Zhou H., Chen H., Fang D. (2018). Preparation and characterization of 3D printed continuous carbon fiber reinforced thermosetting composites. Polym. Test..

[24] Rahman A., Islam Z., Gibbon L., Ulven C.A., La Scala J.J. (2021). 3D printing of continuous carbon fiber reinforced thermoset composites using UV curable resin. Polym. Compos..

[25] Rahman M.A. (2022). Process Optimization of 3D Printing with Continuous Fiber Reinforced UV Curable Thermoset Resin.

[26] Sano Y., Matsuzaki R., Ueda M., Todoroki A., Hirano Y. (2018). 3D printing of discontinuous and continuous fibre composites using stereolithography. Addit. Manuf..

[27] Justo J., Távara L., García-Guzmán L., París F. (2018). Characterization of 3D printed long fibre reinforced composites. Compos. Struct..

[28] Wang X., Jiang M., Zhou Z.W., Gou J.H., Hui D. (2017). 3D printing of polymer matrix composites: A review and prospective. Compos. Part B Eng..

[29] Carneiro O.S., Silva A.F., Gomes R. (2015). Fused deposition modeling with polypropylene. Mater. Des..

[30] Hu W., Chen Y., Lin Y., Xia Q. (2019). Developmental and transcriptomic features characterize defects of silk gland growth and silk production in silkworm naked pupa mutant. Insect Biochem. Mol. Biol..

[31] Ning F., Cong W., Qiu J., Wei J., Wang S. (2015). Additive manufacturing of carbon fiber reinforced thermoplastic composites using fused deposition modeling. Compos. Part B Eng..

[32] Wickramasinghe S., Do T., Tran P. (2020). FDM-Based 3D Printing of Polymer and Associated Composite: A Review on Mechanical Properties, Defects and Treatments. Polymers.

[33] Matsuzaki R., Ueda M., Namiki M., Jeong T.-K., Asahara H., Horiguchi K., Nakamura T., Todoroki A., Hirano Y. (2016). Three-dimensional printing of continuous-fiber composites by in-nozzle impregnation. Sci. Rep..

[34] Shi P., Goh J.C. (2012). Self-assembled silk fibroin particles: Tunable size and appearance. Powder Technol..

[35] O’Connor H.J., Dowling D.P. (2019). Low-pressure additive manufacturing of continuous fiber-reinforced polymer composites. Polymer Composites.

[36] He Q., Wang H., Fu K., Ye L. (2020). 3D printed continuous CF/PA6 composites: Effect of microscopic voids on mechanical performance. Compos. Sci. Technol..

[37] Imeri A., Fidan I., Allen M., A Wilson D., Canfield S. (2018). Fatigue analysis of the fiber reinforced additively manufactured objects. Int. J. Adv. Manuf. Technol..

[38] Gonzalez J.E.A., Wright W.J., Gustinvil R., Celik E. (2022). Hybrid direct ink write 3D printing of high-performance composite structures. Rapid Prototyp. J..

[39] Abdullah A.M., Ding Y., He X., Dunn M., Yu K. (2022). Direct-write 3D printing of UV-curable composites with continuous carbon fiber. J. Compos. Mater..

[40] Peopoly Nylon-like Tough Resin by Peopoly. https://peopoly.net/products/nylon-like-by-peopoly-black-1kg.

[41] Peopoly Deft Resin by Peopoly. https://peopoly.net/products/deft-resin-by-peopoly.

[42] Liqcreate Strong-X. https://www.liqcreate.com/product/strong-x/.

[43] Teijin Tenax Filament Yarn Data-Sheet. https://www.teijincarbon.com/fileadmin/PDF/Datenbl%C3%A4tter_en/Product_Data_Sheet_TSG01en__EU_Filament_.pdf.

[44] Liqcreate Linear Shrinkage of 3D Printing Resin. https://www.liqcreate.com/supportarticles/linear-shrinkage-of-3d-printing-resins/.

[45] Peopoly Peopoly Resin Shrinkage. https://forum.peopoly.net/t/shrinkage-factor/1589.

[46] Aldrich S. Luperox P. Chrome-extension://efaidnbmnnnibpcajpcglclefindmkaj/https://www.sigmaaldrich.com/specification-sheets/166/363/159042-BULK_______ALDRICH__.pdf.

[47] Formlabs Tough 2000. https://formlabs-media.formlabs.com/datasheets/2001340-TDS-ENUS-0P.pdf.

[48] (2020). Standard Test Methods for Density and Specific Gravity (Relative Density) of Plastics by Displacement.

[49] (2015). Standard Test Methods for Constituent Content of Composite Materials.

[50] (2017). Standard Test Method for Tensile Properties of Polymer Matrix Composite Materials.

[51] (2021). Standard Test Method for Flexural Properties of Polymer Matrix Composite Materials.

[52] Ma Y., Yang Y., Sugahara T., Hamada H. (2016). A study on the failure behavior and mechanical properties of unidirectional fiber reinforced thermosetting and thermoplastic composites. Compos. Part B Eng..

[53] Cox H.L. (1952). The elasticity and strength of paper and other fibrous materials. Br. J. Appl. Phys..

[54] Curtin W.A. (1991). Theory of Mechanical Properties of Ceramic-Matrix Composites. J. Am. Ceram. Soc..

[55] Rosen B.W. (1964). Tensile failure of fibrous composites. AIAA J..

[56] Zhou X.-F., Wagner H. (1999). Stress concentrations caused by fiber failure in two-dimensional composites. Compos. Sci. Technol..

[57] Liao K., Reifsnider K.L. (2000). A tensile strength model for unidirectional fiber-reinforced brittle matrix composite. Int. J. Fract..

